# Case Report: Insulin resistance in type 1 diabetes mellitus: the role of genetic factors

**DOI:** 10.3389/fendo.2025.1656453

**Published:** 2025-09-12

**Authors:** Jintao Wei, Shi-Yuan Lu, Tianyue Zhang

**Affiliations:** ^1^ Department of Emergency Medicine, The Second Affiliated Hospital, Zhejiang University School of Medicine, Zhejiang Province Clinical Research Center for Emergency and Critical Care Medicine, Hangzhou, China; ^2^ Department of Gastroenterology, The Second Affiliated Hospital, Zhejiang University School of Medicine, Hangzhou, Zhejiang, China; ^3^ Department of Endocrinology, The Second Affiliated Hospital, Zhejiang University School of Medicine, Hangzhou, Zhejiang, China

**Keywords:** type 1 diabetes, insulin resistance, IGF2BP2, whole-exome sequencing, genetic testing

## Abstract

We report a 52-year-old man with autoantibody-negative type 1 diabetes (T1D) who presented with severe insulin resistance (IR). Whole-exome sequencing (WES) identified a heterozygous mutation in the IGF2BP2 gene (c.248A>G, p. Lys83Thr; rs4402960), associated with type 2 diabetes (T2D) risk. Despite intensive insulin therapy, the patient exhibited markedly elevated insulin requirements (>1.5 U/kg/day; total, 140 U/day) alongside persistent hyperglycemia. The estimated glucose disposal rate (eGDR) was 4.32 mg/kg/min, indicating significant IR. The Somogyi phenomenon was ruled out via continuous glucose monitoring (CGM), and the patient was deemed to have IR. The addition of metformin, acarbose, and dapagliflozin reduced insulin requirements and significantly improved glycemic control. This case suggests that T2D-associated genetic variants may contribute to IR in T1D and underscores the potential value of genetic testing in guiding targeted oral combination therapy.

## Introduction

Type 1 diabetes (T1D) is an autoimmune disease in which the immune system attacks and destroys its own pancreatic cells, leading to abnormal insulin function (1, 2). Traditionally, T1D is defined by insulin deficiency, whereas insulin resistance (IR)—characterized by reduced bodily responsiveness to insulin—is a hallmark feature of type 2 diabetes (T2D). Recently, there has been a growing number of reports on IR in patients with T1D, which impairs glycemic control (3–5). The IGF2BP2 locus (rs4402960) is a well-established genetic risk factor for T2D (6). Functional studies demonstrate that this variant impairs first-phase glucose-stimulated insulin secretion and disrupts insulin signaling pathways (7). However, no reports of its association with T1D have been documented to date.

We report the case of a 52-year-old man with autoantibody-negative T1D who presented with severe IR. The estimated glucose disposal rate (eGDR) was 4.32 mg/kg/min, indicating significant IR with markedly elevated insulin requirements (>1.5 U/kg/day; total, 140 U/day) yet persistent hyperglycemia despite intensive insulin therapy, excluding the Somogyi phenomenon based on continuous glucose monitoring (CGM). Whole-exome sequencing (WES) revealed a heterozygous variant in the IGF2BP2 gene (c.248A>G, p. Lys83Thr; rs4402960), a risk locus for T2D. By adding T2D-related oral medications, especially dapagliflozin, his blood glucose levels were stabilized. This case underscores the clinical relevance of genetic testing in patients with T1D with unexplained IR, as identifying T2D-associated variants may guide targeted therapies and mitigate treatment resistance.

## Case presentation

A 52-year-old man with a 21-year history of T1D was admitted to our department due to uncontrolled blood glucose over the past 21 years. Twenty-one years ago, he presented with unprovoked thirst, frequent urination, fatigue, and weight loss, and was diagnosed with T1D complicated with diabetic ketoacidosis (DKA). He subsequently initiated insulin therapy, which, however, yielded suboptimal results. During the prior hospitalization, T1D was diagnosed based on the patient’s medical history and C-peptide results (five time points: fasting and 30/60/120/180 min post-glucose ingestion, all < 0.01 nmol/L). Though autoantibody-negative, the patient required lifelong insulin from onset and initially presented with DKA, which collectively confirm the T1D diagnosis. The insulin regimen was switched to the standard protocol of insulin glargine and insulin lispro; additionally, dietary/exercise education was provided and CGM was initiated. However, the patient experienced persistent poor glycemic control after discharge, with a maximum total daily insulin dose of 140 units/day, which resulted in readmission. Despite intensive insulin therapy and adherence to dietary and exercise recommendations, his glycemic control remained poor, with persistent hyperglycemia. He also had a long-standing history of pruritus and skin ulcers. His family history was as follows: an aunt with T2D, ex-smoker, 30-year alcohol use, and a history of hypertension.

### Investigation

Physical examination upon this administration showed a body mass index (BMI) of 28.25 kg/m^2^ (height, 178 cm; weight, 89.5 kg), a waist circumference of 99 cm, a blood pressure of 137/80 mmHg, and scattered skin ulcerations detected around the body. Lab examinations showed an elevated HbA1c level of 8.2%, and C-peptide release assay showed exhausted pancreatic islet function (C-peptide levels at fasting and 30/60/120/180 min post-glucose ingestion were all <0.01 nmol/L). Electromyography showed features of diabetic peripheral neuropathy. Abdominal ultrasound showed fatty liver (**Table 1**).

**Table 1:** Clinical and laboratory characteristics.

Clinical features	Result
Sex	Male
Age of onset (years)	31
Course of disease (years)	21
HbA1c%	8.2
C-peptide release assay (nmol/L), fasting and 30/60/120/180 min post-glucose ingestion	<0.01
Electromyography	Features of diabetic peripheral neuropathy
Abdominal ultrasound	Fatty liver
BMI (kg/m^2^)	28.25
Waist (cm)	99
Blood pressure (mmHg)	137/80

The patient’s age at onset (approximately 31 years), presentation with ketoacidosis, and nearly undetectable C-peptide levels are consistent with autoantibody-negative T1D. The patient’s eGDR was 4.32 mg/kg/min, calculated according to the following formula: eGDR (mg/kg/min) = 21.158 − (0.09 × WC)  −  (3.407 × hypertension) − (0.551 × HbA1c), where WC denotes waist circumference (cm), hypertension is coded as yes = 1/no = 0, and HbA1c represents glycated hemoglobin (%) (8). Meanwhile, the patient’s total daily insulin dosage was significantly higher than the estimated dose, and hyperglycemia secondary to hypoglycemia (Somogyi phenomenon) has been ruled out via CGM. Additionally, the patient maintained a stable body weight and exhibited good self-management adherence: he consistently followed a diabetes-specific diet and performed appropriate physical activity, thereby excluding excessive insulin dosage and poor glycemic control caused by binge eating. Thus, IR was suspected in this patient (9). Subsequently, the patient was fully informed of the off-label drug use in this case and signed the informed consent form. Regarding the potential risk of DKA associated with sodium-glucose cotransporter 2 (SGLT-2) inhibitor use in T1D, we repeatedly emphasized that this medication must be administered under the guidance of a diabetologist (10, 11): it should be initiated only when insulin doses are adequate, and during treatment, the patient must comply with regular follow-up visits and periodic ketone monitoring as prescribed to ensure safety. Accordingly, dapagliflozin (5 mg/day) was prescribed. Under careful glucose monitoring and dosage adjustment, the patient’s glycemic control was stabilized. Finally, the maintenance regimen was confirmed as follows: insulin glargine (26 U pre-breakfast, 8 U at bedtime), insulin lispro (15 U–6 U–11 U), plus oral metformin, acarbose, and dapagliflozin. Additionally, the patient was advised to undergo WES prior to discharge.

### Outcome and follow-up

**Figure 1:**
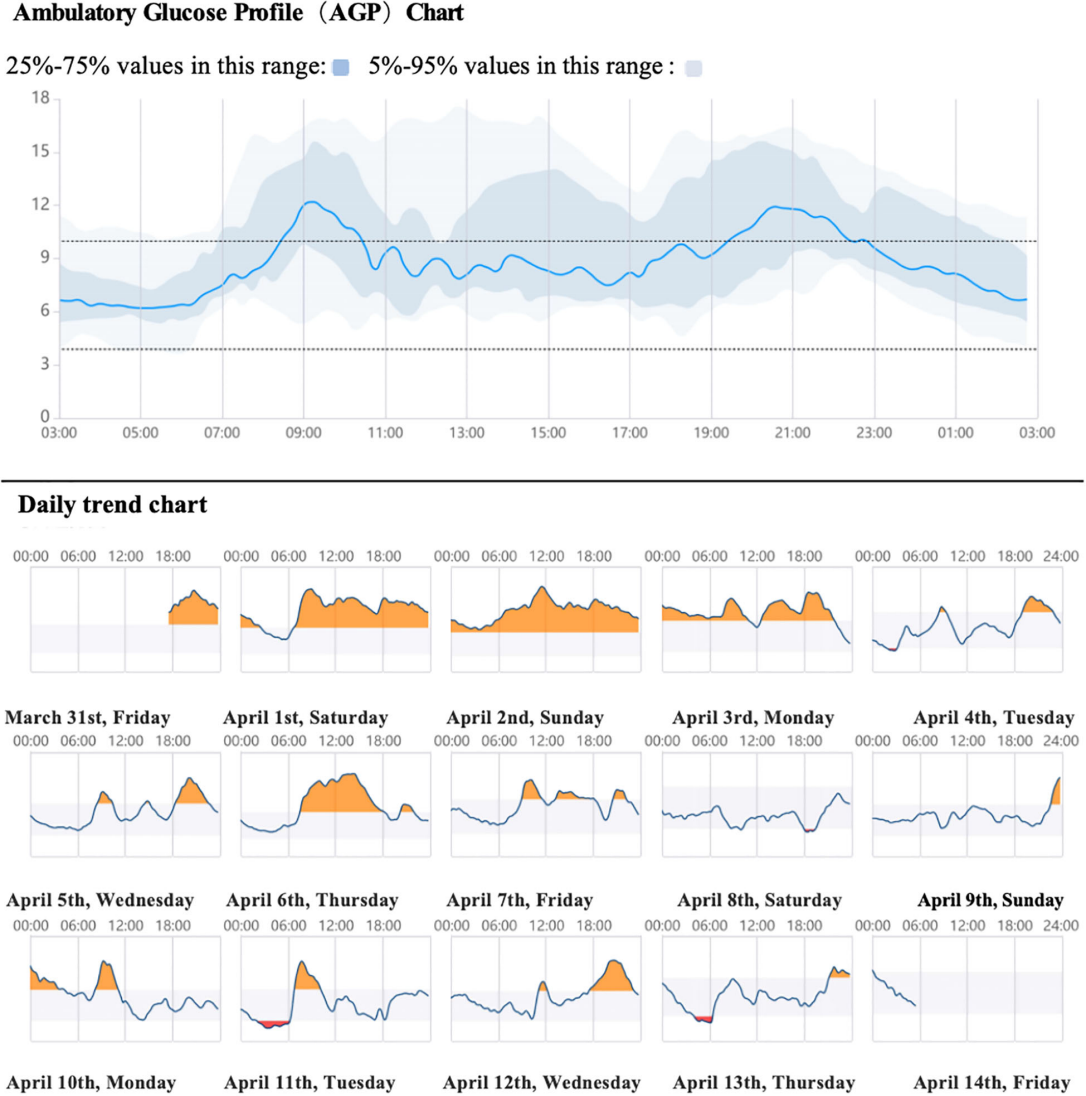
Blood glucose trend chart hospitalization period within 15 days.

**Figure 2:**
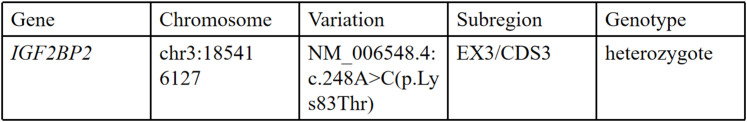
WES sequencing diagram.

The glycemic level of this patient eventually reached the target range and remained stable, and the CGM showed continuous glycemic control in the following days (**Figure 1**). Six months later, his WES report indicated a heterozygous variant in the IGF2BP2 gene (c.248A>G, p. Lys83Thr; rs4402960) (**Figure 2**), a reported risk location of T2DM, which may partially explain his obvious IR.

Additionally, pre-discharge WES was performed: genomic DNA extracted from peripheral blood using a commercial kit, followed by exome enrichment, alignment to GRCh38 (BWA-MEM), variant calling (GATK), and pathogenicity prediction (PolyPhen-2/SIFT).

## Discussion

We report a 52-year-old man with autoantibody-negative T1D who presented with severe IR. Through the combination of metformin, acarbose, and dapagliflozin, his blood glucose was finally stabilized. WES analysis revealed a heterozygous mutation in the IGF2BP2 gene (c.248A>G, p. Lys83Thr; rs4402960), a risk locus for T2D, which may partially explain his pronounced IR. This case highlights the value of genetic testing in T1D with unexplained IR and aligns with prior evidence linking IGF2BP2 polymorphisms to impaired β-cell function and insulin sensitivity in T2D (6, 7).

The hyperinsulinemic–euglycemic clamp (HEC) is the gold standard for evaluating IR, but its application in clinical and research has been limited due to its invasive, time-consuming, and expensive nature (12). Subsequently, the homeostasis model assessment of insulin resistance (HOMA-IR) was developed for clinical research (8). However, HOMA-IR inherently depends on endogenous insulin secretion. Therefore, it was not applicable to the present patient, whose long-term exogenous insulin therapy confounded the method’s reliance on endogenous insulin, rendering the assessment invalid. The eGDR, calculated from readily available clinical parameters such as waist circumference, blood pressure, and glycated hemoglobin, is currently the only practical, non-invasive method for assessing IR in patients with T1D (8, 12). For this patient, the eGDR was 4.32 mg/kg/min and a lower eGDR indicates greater IR. Additionally, we conducted a clinical assessment, which revealed that his total daily insulin dose exceeded 1.5 U/kg/day, with hyperglycemia induced by hypoglycemia ruled out.

It is well known that obesity and weight gain are closely associated with IR in T1D (13, 14). The patient met the criteria for obesity (BMI, 28.25 kg/m^2^; waist circumference, 99 cm). Abdominal fat is a high-risk factor for IR. Therefore, for diabetic obese patients, weight management is also important.

Recent studies have identified that several genetic loci traditionally associated with T2D, such as TCF7L2, IGF2BP2, and FTO, may also influence IR in individuals with T1D, particularly in those with a long disease course or features of metabolic syndrome (15, 16). Furthermore, genetic variants linked to insulin signaling and glucose metabolism—such as polymorphisms in INSR, PPARG, and ADIPOQ—have been associated with increased susceptibility to IR, suggesting a complex interplay between autoimmune and metabolic genetic predispositions in some patients with T1D (17). The WES of this patient indicated heterozygous mutations (c.248A>G, p. Lys83Thr) in the IGF2BP2 gene. This gene mutation is associated with genetic susceptibility to T2D and may exacerbate IR by affecting the insulin signaling pathway. Recent genome-wide association studies have shown that the IGF2BP2 gene promotes the development of T2D by disrupting insulin secretion (18). Groenewoud et al. found that the IGF2BP2 gene reduced glucose-stimulated insulin secretion in the first stage of diabetes development (19). In both Indian and Chinese populations, IGF2BP2 was found to be closely associated with T2D even after adjusting for age, sex, and BMI (20).

For patients with T1D who repeatedly fail to control their condition after insulin therapy, it is necessary to check for IR or the presence of risk genes associated with T2D. Metformin is the first choice of drug therapy for T2D; it reduces blood glucose by reducing liver glucose output and improving peripheral IR, improves multiple cardiovascular risk factors, and improves IR. Metformin is recommended for patients with IR combined with T2D (21). Acarbose is one of the commonly used oral hypoglycemic drugs for T2D and can also be used as an adjuvant therapy for T1D by inhibiting the activity of α-glucosidase in the intestine and delaying the absorption of carbohydrates, thus delaying the increase of postprandial blood glucose (22, 23). Dapagliflozin is a highly selective inhibitor of renal sodium-glucose transporter 2, which inhibits renal glucose reabsorption and increases the excretion of glucose in urine through non-insulin-dependent pathways, thereby improving blood glucose control and reducing body weight (24). Although not part of the standard treatment for T1D, the reasonable use of SGLT-2 can significantly improve blood glucose levels in patients with significant IR, but the risk of ketoacidosis needs to be closely monitored. It should be emphasized that the use of metformin (25), acarbose (26), and SGLT2 inhibitors (dapagliflozin) in patients with T1D is off label. In this case, these agents were introduced under close clinical monitoring and with the patient’s informed consent, given the severe IR and inadequate glycemic control on intensive insulin therapy alone. Notably, when SGLT-2 inhibitors are initiated, attention should be paid to an appropriate reduction in insulin dosage.

This study has some limitations. First, its single-case sample size restricts the generalizability of the findings. Second, the HEC is the gold standard for assessing IR in T1D. However, its clinical implementation remains challenging, making definitive IR diagnosis difficult. Finally, only WES was performed; Sanger validation of the identified IGF2BP2 gene (c.248A>G, p. Lys83Thr; rs4402960) was omitted, potentially compromising the variant’s authenticity confirmation.

## Conclusion

In summary, this case highlights that for patients with T1D with poor glycemic control and marked IR that cannot be fully explained by metabolic factors, genetic testing should be considered. Early identification of relevant genetic variants may help uncover underlying mechanisms, guide the development of individualized treatment strategies, and potentially reduce the risk of complications.

## Data Availability

The raw data supporting the conclusions of this article will be made available by the authors, without undue reservation.
